# Targeting the NLRP3 Inflammasome in Atherosclerosis: A Review of Natural Products and Their Molecular Mechanisms

**DOI:** 10.3390/ijms27083650

**Published:** 2026-04-19

**Authors:** Su-Jin Bae, Hye-Min Seo, Si-Eon You, Jun-Ho Lee

**Affiliations:** Department of Pharmaceutical & Engineering, College of BIT Convergence, Seowon University, Cheongju-si 28674, Republic of Korea; suuujiny03@gmail.com (S.-J.B.); hym912136@gmail.com (H.-M.S.); siun805800@gmail.com (S.-E.Y.)

**Keywords:** atherosclerosis, NLRP3 inflammasome, natural products, vascular inflammation, pyroptosis

## Abstract

Atherosclerotic cardiovascular disease (ASCVD) is increasingly recognized not merely as a lipid-storage disorder but as a chronic, lipid-driven inflammatory condition of the arterial wall. Despite the widespread use of statins and other lipid-lowering therapies, a substantial “residual inflammatory risk” persists, propelling the search for targeted immunopharmacological interventions. At the forefront of this inflammatory cascade is the nucleotide-binding oligomerization domain-like receptor family pyrin domain-containing 3 (NLRP3) inflammasome, which serves as a central orchestrator of vascular inflammation by linking metabolic dysregulation to the innate immune response. Atherogenic danger signals—such as oxidized low-density lipoprotein (ox-LDL) and cholesterol crystals—trigger NLRP3 activation through reactive oxygen species (ROS) generation, lysosomal rupture, and potassium efflux. This, in turn, drives the maturation of pro-inflammatory cytokines (IL-1β and IL-18) and initiates macrophage pyroptosis. In this review, we systematically evaluate the immunomodulatory potential of natural products—both complex extracts and single bioactive compounds—in inhibiting the NLRP3 inflammasome axis. We detail the pharmacological mechanisms by which these natural agents intercept inflammatory signaling at multiple stages: suppressing TLR4/NF-κB-mediated priming, scavenging mitochondrial ROS, and restoring autophagic flux via AMPK/mTOR pathways to prevent inflammasome assembly. By critically analyzing these pathways, we highlight natural product-derived inhibitors as a promising class of immunomodulators capable of attenuating atherosclerotic progression and addressing the persistent challenge of residual inflammatory risk.

## 1. Introduction

Cardiovascular disease (CVD) remains the foremost cause of global morbidity and mortality, presenting a formidable challenge to public health systems in the 21st century [1,2,3]. Driven by aging populations and the escalating prevalence of metabolic risk factors, such as obesity and diabetes, the clinical burden of CVD continues to rise [4,5]. Epidemiological data from the Global Burden of Disease (GBD) Study reveal that prevalent CVD cases nearly doubled from 271 million in 1990 to 523 million in 2019, with ischemic heart disease and stroke serving as the primary contributors [1,6]. Furthermore, current projections indicate that without innovative therapeutic interventions, the economic toll of CVD in the United States alone could exceed $1.1 trillion by 2035 [7,8].

Historically conceptualized as a passive lipid-storage disorder characterized by cholesterol accumulation in the arterial intima [9,10], atherosclerosis is now fundamentally recognized as a chronic, low-grade inflammatory condition orchestrated by both innate and adaptive immune responses [11,12,13]. The atherogenic process initiates with endothelial dysfunction—precipitated by disturbed blood flow or systemic risk factors—which upregulates adhesion molecules such as VCAM-1 and ICAM-1 [14,15]. This endothelial activation facilitates the transmigration of monocytes, which subsequently differentiate into macrophages, engulf oxidized low-density lipoprotein (ox-LDL), and transform into foam cells, thereby establishing the hallmark of early atherosclerotic lesions [16,17,18].

While lipid-lowering pharmacotherapies, particularly HMG-CoA reductase inhibitors (statins) and PCSK9 inhibitors, remain the cornerstone of ASCVD management, they do not entirely abrogate cardiovascular risk [19,20]. Major clinical trials, including PROVE-IT and IMPROVE-IT, have clearly demonstrated that a significant “residual inflammatory risk” persists even when optimal LDL-cholesterol levels are achieved [21,22,23]. The landmark Canakinumab Anti-inflammatory Thrombosis Outcome Study (CANTOS) provided definitive proof-of-concept for the inflammatory hypothesis, revealing that neutralizing interleukin-1β (IL-1β) significantly attenuates cardiovascular event rates independent of lipid reduction [24]. Moreover, the recent LoDoCo2 (Low-Dose Colchicine 2) trial further validated the clinical efficacy of targeting inflammation in chronic coronary disease [25,26].

At the molecular epicenter of this inflammatory storm lies the nucleotide-binding oligomerization domain-like receptor family pyrin domain-containing 3 (NLRP3) inflammasome [27]. Unlike other pattern recognition receptors (PRRs) that primarily detect microbial pathogens, NLRP3 functions as a unique sensor of sterile cellular stress [28,29]. During atherogenesis, endogenous danger signals—such as cholesterol crystals, ox-LDL, and intracellular calcium influx—trigger the assembly of the NLRP3 inflammasome complex, which comprises the NLRP3 sensor, the adaptor protein ASC, and pro-caspase-1 [30,31,32]. This macromolecular assembly culminates in the activation of caspase-1, which subsequently catalyzes the proteolytic cleavage of pro-IL-1β and pro-IL-18 into their mature, bioactive forms, while also executing pyroptosis, a lytic form of programmed cell death that potently amplifies vascular inflammation [33,34,35].

Despite the clear therapeutic promise of deactivating the NLRP3 pathway, current pharmacological options exhibit notable limitations. Biologic agents like canakinumab are associated with prohibitive costs and an increased risk of fatal infections, whereas broad-spectrum anti-inflammatory agents like methotrexate failed to confer cardiovascular benefits in the CIRT trial, underscoring the necessity for highly specific, pathway-targeted interventions [24,36]. Consequently, there is an urgent need for novel, safe, and cost-effective NLRP3 inhibitors. Historically, natural products have served as a prolific reservoir for drug discovery, with over 50% of approved pharmaceuticals being derived from or inspired by natural sources [37,38,39]. Unlike synthetic single-target drugs, natural phytochemicals—including polyphenols, alkaloids, and terpenes—frequently exhibit “multi-target” immunopharmacology. They can simultaneously suppress upstream priming signals (e.g., TLR4/NF-κB) and disrupt downstream inflammasome assembly, while actively reinforcing cellular antioxidant defenses via the Nrf2 pathway [40,41,42,43].

In this review, we provide a comprehensive immunopharmacological analysis of the NLRP3 inflammasome’s role in atherosclerosis and systematically evaluate recent preclinical evidence regarding natural products that selectively target this axis. By dissecting these immunomodulatory mechanisms, we highlight the potential of these natural agents to bridge the therapeutic gap in current ASCVD management and effectively address residual inflammatory risk.

## 2. Pathophysiological Framework of Atherosclerosis and Inflammasome Biology

### 2.1. Pathogenesis of Atherosclerosis: From Endothelial Dysfunction to Plaque Instability

Contemporary cardiovascular immunology redefines atherosclerosis not simply as a passive disorder of lipid storage, but rather as a chronic, non-resolving inflammatory pathology driven by a complex, dynamic interplay among retained lipoproteins, the vascular endothelium, and the innate immune system [11,12]. This pathological cascade fundamentally originates with endothelial dysfunction, typically precipitated by altered hemodynamic forces—such as disturbed laminar shear stress at arterial bifurcations—coupled with systemic metabolic risk factors like hypertension, hyperglycemia, and dyslipidemia [14,44]. Such initial insults compromise endothelial barrier integrity, triggering the robust upregulation of critical adhesion molecules, notably vascular cell adhesion molecule-1 (VCAM-1) and intercellular adhesion molecule-1 (ICAM-1) [45,46]. These molecular shifts orchestrate the initial tethering, rolling, and subsequent firm adhesion of circulating monocytes to the inflamed intima, representing the critical initiating event in atherogenesis [47,48].

Concurrently, circulating low-density lipoprotein (LDL) particles breach the subendothelial space, where they become tethered to extracellular matrix proteoglycans [49,50]. Sequestered within this microenvironment, LDL undergoes extensive enzymatic and oxidative modifications—mediated largely by myeloperoxidase and reactive oxygen species (ROS)—transforming into highly immunogenic oxidized LDL (ox-LDL) [10,30]. Acting as a potent damage-associated molecular pattern (DAMP), ox-LDL potently induces the local innate immune response. Once infiltrated, monocytes differentiate into tissue macrophages and avidly internalize these modified lipids via specific scavenger receptors, predominantly CD36, SR-A1, and LOX-1 [51,52,53]. Because these receptors bypass classical negative feedback regulation, macrophages relentlessly engulf lipids, ultimately transforming into lipid-laden foam cells [9,17].

As the atheromatous lesion progresses, the unrelenting intracellular lipid burden severely overwhelms macrophage metabolic capacity, precipitating profound endoplasmic reticulum (ER) stress and lysosomal failure [54,55]. This sustained cellular distress cultivates an aggressively pro-inflammatory microenvironment that stimulates the migration of vascular smooth muscle cells (VSMCs) from the medial layer into the intima [56,57]. While these VSMCs initially attempt to stabilize the growing plaque by depositing a collagen-rich fibrous cap, the chronic inflammatory assault eventually drives VSMC apoptosis and accelerates matrix metalloproteinase (MMP)-dependent collagen degradation [58,59]. This pathological sequence culminates in the expansion of a necrotic core—replete with apoptotic debris and cholesterol crystals—that critically compromises plaque architectural integrity, predisposing it to catastrophic rupture and acute thrombotic occlusion [29,60,61].

### 2.2. Molecular Mechanisms of NLRP3 Inflammasome Activation

The innate immune system relies heavily on PRRs to surveil the microenvironment for metabolic danger signals. Among these, the NLRP3 inflammasome has distinguished itself as the paramount intracellular sensor governing sterile vascular inflammation [27,62]. Structurally, the NLRP3 protein is composed of a C-terminal leucine-rich repeat (LRR) domain responsible for ligand sensing, a central NACHT domain harboring the ATPase activity essential for oligomerization, and an N-terminal pyrin domain (PYD) that facilitates homotypic interactions with the adaptor protein ASC [28,63]. To prevent aberrant autoinflammation, the activation of the NLRP3 inflammasome is strictly regulated by a two-step regulatory checkpoint mechanism.

The initial step, conventionally recognized as the “priming” signal (Signal 1), involves the transcriptional licensing of inflammasome components [64]. Upon the recognition of pathogen-associated molecular patterns (PAMPs) or pro-inflammatory cytokines (e.g., TNF-α, IL-1β) by Toll-like receptors (TLRs), the NF-κB signaling cascade is potently activated [65,66]. This cascade drives the nuclear translocation of NF-κB subunits, inducing the rapid transcriptional upregulation of NLRP3 and pro-IL-1β, which are otherwise maintained at rate-limiting trace levels in resting macrophages [67,68]. Furthermore, acute priming can be expedited via post-translational modifications, prominently the deubiquitination of NLRP3, empowering the immune cell to rapidly counter acute metabolic stress [69].

Following successful priming, a distinct second stimulus (Signal 2) triggers the macromolecular assembly of the inflammasome complex. Given the vast structural diversity of known NLRP3 activators, the receptor is hypothesized to sense a convergent downstream cellular disturbance rather than directly binding specific ligands [70]. A primary mechanistic trigger is the perturbation of intracellular ion homeostasis, particularly potassium (K+) efflux driven by P2X7 purinergic receptor activation or pore-forming toxins, which acts as an indispensable prerequisite for NLRP3 oligomerization [71,72]. In parallel, lysosomal destabilization serves as a robust trigger; the frustrated phagocytosis of crystalline matter induces lysosomal rupture, spilling Cathepsin B into the cytosol to directly instigate NLRP3 assembly [73]. Additionally, severe mitochondrial dysfunction actively feeds this cycle by generating copious mitochondrial ROS (mtROS) and discharging oxidized mitochondrial DNA (mtDNA) into the cytoplasmic space [74,75]. Finally, NIMA-related kinase 7 (NEK7) binds securely to the NLRP3 LRR domain, functioning as a vital regulatory partner that licenses the oligomerization of the sensor [76]. Once fully assembled, the NLRP3-ASC complex recruits pro-caspase-1, initiating its autocatalytic cleavage into highly active caspase-1 [77].

### 2.3. The Role of NLRP3 Inflammasome in Atherosclerotic Progression

Within the specific immunopathological context of atherosclerosis, the NLRP3 inflammasome operates as the central orchestrator seamlessly bridging systemic lipid dysregulation with localized vascular inflammation [31]. In the atheromatous core, cholesterol crystals and oxidized LDL dominate as the primary endogenous activators. As macrophage foam cells become fatally engorged with lipids, unesterified cholesterol precipitates into sharp crystalline structures. The subsequent phagocytosis of these crystals mechanically perforates the lysosomal membrane, triggering Cathepsin B leakage—a potent, direct signal for profound NLRP3 activation [29,73]. Concurrently, ox-LDL exerts a dual pathogenic role: it primes the inflammasome via CD36-TLR4 signaling while simultaneously driving its activation through intracellular crystallization and ROS hypersecretion [30,74]. Furthermore, turbulent hemodynamic shear stress at arterial bifurcations directly upregulates endothelial NLRP3 expression via sterol regulatory element-binding proteins (SREBPs), establishing focal epicenters of vascular inflammation [78].

**Figure 1:**
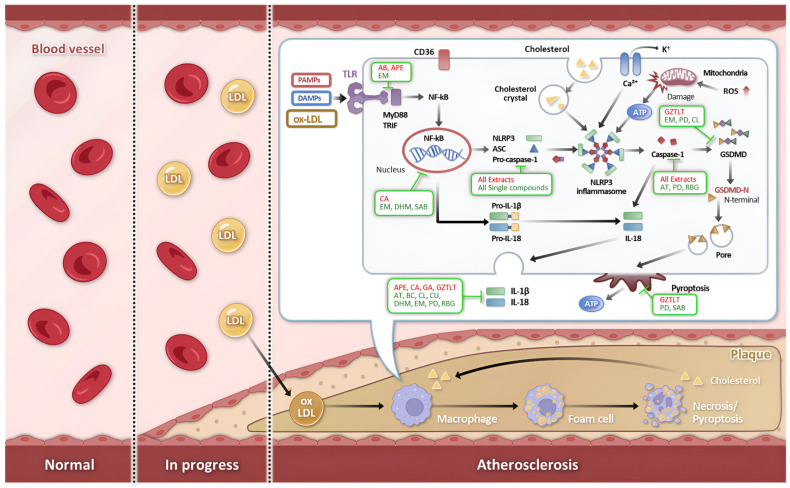
Schematic diagram of atherosclerosis progression and the potential therapeutic interventions by natural products. The pathogenesis of atherosclerosis is driven by endothelial dysfunction and the accumulation of damage-associated molecular patterns (DAMPs) such as oxidized low-density lipoprotein (ox-LDL) and cholesterol crystals. These danger signals trigger NLRP3 inflammasome activation in macrophages, leading to the maturation and secretion of pro-inflammatory cytokines (IL-1β and IL-18), which subsequently promotes foam cell formation and plaque progression. The investigated natural agents, categorized into complex extracts and single bioactive compounds, exert anti-atherosclerotic effects by intercepting multiple stages of this inflammatory cascade. Their mechanistic targets include the inhibition of the upstream NF-κB priming signal, scavenging of mitochondrial reactive oxygen species (ROS), restoration of autophagy via the AMPK/mTOR pathway, and direct blockade of NLRP3 inflammasome assembly.

The pharmacological consequences of NLRP3 activation within the arterial wall are aggressively multifaceted. Active caspase-1 catalytically cleaves pro-IL-1β and pro-IL-18 into their mature, highly bioactive forms. Secreted IL-1β dramatically amplifies the regional inflammatory cascade by upregulating endothelial adhesion molecules and chemokines, thereby fueling a relentless feed-forward loop of leukocyte recruitment [31,67]. In parallel, IL-18 synergizes with local cytokines to drive interferon-gamma (IFN-γ) production, which directly impairs collagen synthesis by VSMCs, precipitating fibrous cap thinning [59]. Crucially, caspase-1 also executes the cleavage of Gasdermin D (GSDMD), releasing its lipophilic N-terminal domain to assemble lethal pores within the plasma membrane. This execution pathway, defined as pyroptosis, drives inflammatory lytic cell death, effectively disgorging intracellular DAMPs and lipid contents to massively expand the necrotic core and ultimately destabilize the atherosclerotic plaque [33]. The overall progression of atherosclerosis, highlighting the pathological mechanisms of NLRP3 inflammasome activation by danger signals, its central contribution to the inflammatory cascade, and the specific regulatory points targeted by various natural bioactive agents, is schematically shown in Figure 1.

## 3. Natural Products Targeting NLRP3 Inflammasome in Atherosclerosis: Preclinical Evidence

The immunopharmacological landscape for atherosclerotic cardiovascular disease has been substantially broadened by the discovery of natural products uniquely capable of modulating the NLRP3 inflammasome signaling axis. Derived from phylogenetically diverse botanical and fungal sources, these agents demonstrate remarkable mechanistic sophistication, effectively intercepting vascular inflammation through multi-level inflammasome regulation. The accumulating preclinical evidence can be systematically categorized into complex natural extracts—which leverage synergistic interactions among multiple bioactive constituents—and purified single-molecule compounds, each providing distinct strategic advantages for targeted therapeutic development. The preclinical evidence for each natural product, highlighting their specific inhibitory roles within the NLRP3 signaling pathway as illustrated in Figure 1, is discussed in detail in the following sections.

### 3.1. Natural Product Extract Targeting NLRP3 Inflammasome

Complex natural product extracts represent a highly promising class of anti-atherosclerotic agents, delivering synergistic, multi-target immunomodulation through the orchestrated pharmacological actions of diverse bioactive constituents. These therapeutic formulations encompass both historically validated traditional herbal preparations and modern standardized botanical extracts, all of which are increasingly subjected to rigorous phytochemical characterization and deep mechanistic scrutiny. The preclinical evidence for natural product extracts that have demonstrated NLRP3 inflammasome-targeting anti-inflammatory effects in atherosclerosis models over the past five years is comprehensively summarized in Table 1.

#### 3.1.1. Agaricus Bisporus Extract (AB)

Agaricus bisporus, a globally consumed culinary mushroom, harbors potent bioactive components including beta-glucan, ergothioneine, and various phenolic compounds, which are widely recognized for their robust antioxidative and immunomodulatory properties. A recent preclinical study evaluated its therapeutic efficacy in LDLR^−/−^ mice subjected to a high-fat diet (HFD). Administration of the AB extract (7.5 g/kg) not only corrected systemic metabolic derangements—curbing body weight gain and hyperglycemia—but also profoundly attenuated atherosclerotic plaque formation. Mechanistically, the AB extract deployed a multi-targeted immunometabolic strategy. It significantly upregulated GATA4 and PPARα, critical transcription factors governing hepatic lipid homeostasis and autophagic flux. Concurrently, it abrogated the expression of TLR4 alongside key pro-inflammatory mediators such as TNF-α and iNOS. This sophisticated orchestration of metabolic and immune nodes led to a dramatic reduction in both NLRP3-positive cellular infiltration and cleaved caspase-3 activity within the atheromatous lesions, firmly suggesting that Agaricus bisporus inhibits the NLRP3 inflammasome by neutralizing upstream oxidative stress and resolving metabolic dysregulation [79].

#### 3.1.2. Apple Polyphenol Extract (APE)

Apple Polyphenol Extract (APE), predominantly comprising chlorogenic acid, phlorizin, and procyanidin B2, has exhibited formidable cardioprotective properties. Utilizing an established model of atherosclerosis in LDLR^−/−^ mice fed a high-fat, high-cholesterol diet (HFCD), researchers demonstrated that oral gavage of APE (125 and 500 mg/kg/day) dose-dependently restrained aortic intimal thickening and blunted inflammatory macrophage infiltration. The pronounced anti-atherosclerotic efficacy of APE was mechanistically mapped to its potent pharmacological blockade of the TLR4-driven inflammatory cascade. Protein analysis confirmed that APE robustly suppressed the activation of the TLR4/MyD88/NF-κB signaling axis, thereby blocking the essential “priming” signal required for inflammasome licensing. Consequently, the protein expression of NLRP3, ASC, and cleaved caspase-1 was sharply curtailed, effectively preventing the subsequent maturation and release of IL-1β and IL-18. These findings establish APE as a powerful immunomodulator capable of dismantling the atherogenic inflammatory loop at its earliest upstream signaling phase [80].

#### 3.1.3. Crataegus Aronia Extract (CA)

Crataegus aronia, an ethnobotanical agent traditionally employed in the management of hypertension and cardiovascular conditions, possesses a rich profile of active flavonoids and proanthocyanidins. In a Wistar rat model of HFD-induced atherosclerosis, CA intervention (200 mg/kg/day) substantially rectified dyslipidemia, markedly lowering circulating LDL-C while elevating protective HDL-C. Histopathological evaluations highlighted a significant reduction in intimal fat deposition and aortic foam cell entrapment. From a mechanistic standpoint, CA specifically targets the destructive oxidative stress-inflammation axis. By restoring the catalytic activity of endogenous antioxidant scavengers—including SOD, CAT, and GSH-Px—CA effectively quenched intracellular ROS accumulation. This potent antioxidative tone directly facilitated the suppression of NF-κB p65 phosphorylation, subsequently crippling the downstream expression of NLRP3, ASC, and cleaved caspase-1. Ultimately, CA attenuates vascular inflammation through the dual regulation of ROS-dependent inflammasome activation and consequential pro-inflammatory cytokine secretion [81].

#### 3.1.4. Ginkgo Flavone Aglycone (GFA)

Ginkgo Flavone Aglycone (GFA) represents the purified, bioactive flavonol cores isolated following the hydrolysis of Ginkgo biloba extracts. In ApoE^−/−^ mice challenged with an atherogenic diet, oral administration of GFA (30 and 90 mg/kg/day) significantly hindered the progression of plaque formation across both the thoracic and abdominal aorta. The immunopharmacological action of GFA is intimately tied to its targeted activation of the Nrf2 signaling cascade. Treatment with GFA markedly promoted the nuclear translocation of Nrf2, thereby upregulating the critical antioxidant enzyme Heme Oxygenase-1 (HO-1) and driving a sharp decline in vascular ROS burden. By actively fortifying the endothelial and macrophage antioxidant defenses, GFA thereby removing the critical trigger for NLRP3 inflammasome assembly and preventing endothelial pyroptosis and suppressing systemic levels of mature IL-1β and IL-18 [82].

#### 3.1.5. Guizhitongluo Tablet (GZTLT)

Guizhitongluo Tablet (GZTLT) is an esteemed traditional Chinese botanical formulation comprising Cinnamomum cassia, Sargassoaceae, and Ilex angustifolia. In ApoE^−/−^ murine models, therapeutic intervention with GZTLT dose-dependently restricted aortic plaque expansion and necrotic core volume, while simultaneously promoting plaque stability via augmented collagen deposition. A groundbreaking mechanistic insight from this study was the identification of the mechanosensitive ion channel Piezo1 as a direct pharmacological target of GZTLT. The formulation successfully inhibited Piezo1-gated calcium (Ca^2+^) influx, blocking an essential biophysical signal required for NLRP3 complex assembly. In targeted validation experiments utilizing the selective Piezo1 agonist Yoda1, GZTLT effectively blocked the anomalous Ca^2+^ entry, subsequently repressing the protein expression of NLRP3, GSDMD, and cleaved caspase-1. These compelling data illustrate that GZTLT halts atherogenesis by severing the pathological Piezo1-Ca^2+^-NLRP3 axis, thereby shielding vascular cells from pyroptotic death [83].

**Table 1:** Summary of extracts targeting the NLRP3 inflammasome in atherosclerosis.

Natural Product	Source/Origin	Experimental Model	Dosage and Administration	Key Mechanisms and Targets	Primary Outcomes	Ref.
Agaricus Bisporus (AB)	Edible mushroom	LDLR^−/−^ mice, HFD	Drinking water in 12 weeks 7.5 g/kg	↓ MOMA-2, TLR4, NLRP3, cle-caspase-3 ↓ COL1A1, TNF-α, SOD1, iNOS, GPX3 ↑ GATA4, PPARα	Decreased blood sugar Decreased serum AST activity	[79]
Apple Polyphenol Extract (APE)	*Malus domestica*	LDLR^−/−^ mice, HFCD	Oral administration 8 weeks 125, 500 mg/kg/day	↓ TLR4 signaling pathway (NF-κB, MyD88, TRIF, IKKβ), NLRP3, ASC, cle-caspase-1 ↓ IL-1β, IL-18	Decreased atherosclerotic necrosis Decreased foam cell infiltration Increased HDL-C	[80]
Crataegus Aronia (CA)	Hawthorn berries	Wistar Rats, HFD	Oral administration 4 weeks 200 mg/kg/day	↓ NF-κB p65 nuclear translocation, NF-κB activation, NLRP3, ASC, cle-caspase-1 ↓ IL-1β, IL-18 ↓ Intracellular ROS levels	Decreased TC, TG and LDL-C Reduced intimal thickening and lipid Accumulation Increased HDL-C and Cell Viability	[81]
Ginkgo Flavone Aglycone (GFA)	*Ginkgo biloba* leaves	ApoE^−/−^ mice, HFD	Oral administration 12 weeks 30, 90 mg/kg/day	↓ KEAP1, NLRP3, cle-caspase-1 ↓ IL-1β, IL-18 ↓ Intracellular ROS levels ↑ Nrf2, HO-1	Decreased TC, TG and LDL-C Increased serum HDL-C	[82]
Guizhi Tongluo Tablet (GZTLT)	Herbal formula *	ApoE^−/−^ mice, HFD	Oral administration 8 weeks 0.52, 1.04, 2.08 g/kg/day	↓ NLRP3 inflammasome adapter protein (NLRP3, ASC, cle-caspase-1), GSDMD, Pyroptosis ↓ IL-1β ↓ Piezo1-mediated Ca^2+^ influx	Decreased lipid levels Decreased Foam cell expression	[83]

* Guizhitongluo Tablet consists of *Cinnamomum cassia*, *Sargassoaceae*, and *Ilex angustifolia*. ↑, upregulation or activation; ↓, downregulation of inhibition. AB, *Agaricus bisporus*; cle-caspase, cleaved caspase; APE, Apple polyphenol extract; ASC, Apoptosis-associated speck-like protein containing a CARD; CA, *Crataegus aronia*; GFA, Ginkgo flavone aglycone; GSDMD, Gasdermin D; GZTLT, Guizhitongluo tablet; HFCD, High-fat, high-cholesterol diet; HFD, High-fat diet; HO-1, Heme oxygenase-1; IL, Interleukin; NF-κB, Nuclear factor-kappa B; NLRP3, NOD-like receptor family pyrin domain containing 3; Nrf2, Nuclear factor erythroid 2-related factor 2; PPARα, Peroxisome proliferator-activated receptor alpha; ROS, Reactive oxygen species; SOD1, Superoxide dismutase 1; TLR4, Toll-like receptor 4; TNF-α, Tumor necrosis factor-alpha.

### 3.2. Single Bioactive Compounds Targeting NLRP3 Inflammasome

While multi-component extracts provide profound synergistic benefits, the isolation and characterization of single bioactive compounds enable the precise elucidation of molecular target engagement and ensure higher reproducibility in clinical translations. A diverse array of secondary metabolites—ranging from terpenoids to flavonoids and stilbenes—has emerged as potent, targeted inhibitors of the NLRP3 inflammasome. These molecules selectively interrupt specific critical nodes within the activation cascade, including the quenching of mitochondrial ROS, the induction of autophagic flux, and the direct antagonism of upstream receptor signaling. The preclinical evidence for single bioactive compounds that have demonstrated NLRP3 inflammasome-targeting anti-inflammatory effects in atherosclerosis models over the past five years is comprehensively summarized in Table 2.

#### 3.2.1. Artemisinin

Artemisinin, an internationally recognized sesquiterpene lactone isolated from Artemisia annua, exerts striking anti-atherosclerotic efficacy in ApoE^−/−^ mice maintained on a high-fat diet. The oral administration of artemisinin (50 and 100 mg/kg) markedly contracted atherosclerotic lesion areas while correcting systemic hyperlipidemia by lowering total cholesterol (TC) and triglyceride (TG) levels. Mechanistically, artemisinin initiates its immunomodulatory effects within aortic macrophages by potently activating AMP-activated protein kinase (AMPK). This critical kinase activation imposes a strict suppressive effect on the NF-κB signaling pathway, subsequently repressing the protein expression of NLRP3, ASC, and cleaved caspase-1. Additionally, artemisinin successfully downregulated the endothelial presentation of critical vascular cell adhesion molecules (VCAM-1 and ICAM-1), effectively disrupting monocyte–endothelial crosstalk and curbing early foam cell formation [84].

#### 3.2.2. Baicalin

Baicalin, a predominant flavone glucuronide derived from Scutellaria species, exhibits formidable dual anti-inflammatory and antioxidant activities within the atherosclerotic microenvironment. In ApoE^−/−^ mice, therapeutic intervention with baicalin (50 and 100 mg/kg) profoundly minimized plaque area and halted aggressive lesion progression. Its protective immunopharmacology revolves around the robust suppression of reactive oxygen species (ROS) alongside the targeted inhibition of both the NF-κB and MAPK signaling cascades. This multifaceted molecular interference culminated in the marked downregulation of NLRP3 and caspase-1 at both the transcriptional and translational levels. Consequently, baicalin drastically attenuated the release of mature IL-1β and IL-18 while dampening the expression of cellular adhesion molecules, effectively neutralizing the pervasive oxidative and inflammatory stress in the vascular wall [85].

#### 3.2.3. Corilagin

Corilagin, a highly active polyphenol isolated from Phyllanthus species, demonstrates an innovative mechanism of alleviating atherosclerosis by selectively antagonizing the olfactory receptor 2 (Olfr2) signaling pathway. In ApoE^−/−^ mice exposed to a high-fat, high-cholesterol diet, the administration of corilagin (40 mg/kg) significantly repressed the expression of Olfr2 and its direct downstream effector, Adcy3. This targeted pharmacological suppression dismantled the signaling infrastructure necessary for NLRP3 inflammasome activation, leading to significantly reduced levels of NLRP3, cleaved caspase-1, NEK7, and ASC. Furthermore, corilagin actively orchestrated the immunometabolic reprogramming of macrophages, driving polarization away from the inflammatory M1 phenotype toward the reparative M2 phenotype, while concurrently correcting systemic lipid profiles by reducing TG, TC, and LDL-C concentrations [86].

#### 3.2.4. Curcumin

Curcumin, the principal bioactive polyphenol extracted from Curcuma longa, serves as a well-documented antagonist of the NLRP3 inflammasome in the context of cardiovascular disease. Utilizing C57BL/6 mice challenged with a high-fat diet, treatment with curcumin (100 mg/kg) induced a profound reduction in total atherosclerotic plaque area and structurally preserved fibrous cap thickness. Mechanistically, curcumin directly suppressed the protein synthesis of critical inflammasome components, notably NLRP3 and ASC, effectively shutting down the secretion of mature IL-1β and TNF-α. In addition to its anti-inflammatory prowess, curcumin optimized lipid metabolism and rescued endothelial function by significantly elevating local nitric oxide (NO) bioavailability. These results underscore curcumin’s capacity to impart broad vascular protection by intercepting the physical assembly and downstream activation of the NLRP3 complex [87].

#### 3.2.5. Dihydromyricetin (DHM)

Dihydromyricetin (DHM), a prominent bioactive flavonoid, actively mitigates atherogenesis by simultaneously promoting protective mitophagy and actively silencing the NF-κB/NLRP3 inflammatory axis. In ApoE^−/−^ models, DHM delivery (50 and 200 mg/kg) remarkably suppressed lipid deposition and advanced plaque formation. At the molecular level, DHM forcefully upregulated the expression of essential mitophagy-related sentinels (PINK1 and Parkin) while increasing the LC3-II/LC3-I ratio. This targeted promotion of autophagic flux accelerated the clearance of critically damaged mitochondria, effectively stripping the cell of excess ROS production. Concurrently, DHM hindered the phosphorylation and subsequent nuclear entry of NF-κB p65, directly suppressing the de novo synthesis of NLRP3 and caspase-1. This elegant dual mechanism ensures the comprehensive blockade of inflammasome activation and its associated pro-inflammatory cytokine production [88].

#### 3.2.6. Emodin

Emodin, a structurally distinct anthraquinone derivative, acts as a potent pharmacological inhibitor of atherosclerotic inflammation by specifically targeting the TLR4/MyD88/NF-κB signaling axis. In ApoE^−/−^ mice, emodin therapy (10 and 20 mg/kg) elicited a significant contraction of aortic lesion size. Sophisticated molecular docking and co-immunoprecipitation analyses revealed that emodin physically binds to both TLR4 and its crucial adaptor MyD88, thereby sterically disrupting their functional interaction. This upstream receptor blockade effectively suppressed the downstream NF-κB cascade, precipitating a steep decline in the transcription of NLRP3 and pro-IL-1β. Furthermore, emodin demonstrated a remarkable capacity to inhibit Gasdermin D (GSDMD)-driven pyroptosis, introducing a novel, highly effective immunopharmacological strategy for preserving cellular integrity and stabilizing vulnerable atherosclerotic plaques [89].

#### 3.2.7. Polydatin

Polydatin, the natural glycoside precursor of resveratrol, actively attenuates the progression of atherosclerosis by repairing stalled autophagic flux through targeted mTOR inhibition. In ApoE^−/−^ mice, treatment with polydatin (50, 100, and 200 mg/kg) achieved a striking, dose-dependent regression of lipid deposition and total plaque area. Mechanistically, polydatin forcefully inhibited the pathological phosphorylation of mTOR and p62, simultaneously elevating the LC3-II/LC3-I ratio—a hallmark of reactivated autophagy. This pharmacological restoration of autophagic clearance efficiently swept away aggregated NLRP3 inflammasome components and halted the progression of pyroptosis, a finding corroborated by significant reductions in cleaved GSDMD-N levels and TUNEL-positive apoptotic cells. Moreover, polydatin fortified plaque architecture by actively promoting the accumulation of stabilizing collagen and smooth muscle cells [90].

#### 3.2.8. Resibufogenin

Resibufogenin, a highly potent bioactive compound isolated from traditional toad venom extracts, exhibits an extraordinary capacity to directly disrupt the structural assembly of the NLRP3 inflammasome. In ApoE^−/−^ mice, the administration of resibufogenin at remarkably low doses (1, 3, and 5 mg/kg) rapidly shrank plaque dimensions and normalized severe dyslipidemia. Exploring its precise molecular engagement, researchers discovered that resibufogenin non-covalently binds directly to the critical Cys-279 residue within the NACHT domain of the NLRP3 protein. This direct physical interaction sterically hindered NLRP3 oligomerization and entirely blocked its necessary docking with the ASC adaptor. By executing this direct structural blockade, resibufogenin bypassed upstream priming pathways to conclusively arrest caspase-1 activation and halt IL-1β/IL-18 secretion, presenting a highly specific mechanism for alleviating macrophage infiltration and vascular inflammation [91].

#### 3.2.9. Salvianolic Acid B (SAB)

Salvianolic acid B (SAB), a major hydrophilic constituent extracted from the roots of Salvia miltiorrhiza, offers robust cardiovascular protection by strictly regulating the NF-κB/NLRP3 signaling network. In LDLR^−/−^ murine models, SAB administration (12.5, 25, and 50 mg/kg) generated a sharp, dose-dependent decline in lipid accumulation and overall plaque expansion. Pharmacologically, SAB completely neutralized HFD-induced ROS overproduction and blocked the nuclear translocation of the NF-κB p65 subunit. This resulting transcriptional silencing forced a dramatic reduction in both NLRP3 sensor expression and the synthesis of downstream pro-inflammatory cytokines. Detailed histological assessments further confirmed a marked decrease in foam cell retention coupled with enhanced collagen deposition, highlighting SAB’s critical role in stabilizing atheromatous lesions through powerful immunomodulation [92].

#### 3.2.10. Scutellarin

Scutellarin, a therapeutic flavonoid, introduces a unique immunopharmacological mechanism by actively preventing vascular smooth muscle cell (VSMC)-derived foam cell formation via the promotion of autophagy. In ApoE^−/−^ mice subjected to scutellarin treatment (30 mg/kg), both the severity of aortic lesions and the extent of localized lipid accumulation were profoundly curtailed. Mechanistically, scutellarin effectively restored stalled autophagic flux—a process typically paralyzed by lipotoxic agents such as oleic acid—thereby accelerating the cellular clearance of the intracellular lipid burden. This restoration of metabolic homeostasis inherently removed the stress signals required to activate the NLRP3 inflammasome, translating into drastically reduced protein expression of NLRP3 and ASC. By actively suppressing NLRP3-mediated pyroptosis and lipid engorgement specifically within the VSMC population, scutellarin provides a highly specialized and innovative therapeutic avenue for maintaining plaque stability [93].

**Table 2:** Summary of single bioactive compounds targeting the NLRP3 inflammasome in atherosclerosis.

Single Compound	Source/Origin	Experimental Model	Dosage and Administration	Key Mechanisms and Targets	Primary Outcomes	Ref.
Artemisinin (AT)	*Artemisia annua* L.	ApoE^−/−^ mice, HFD	Oral administration 8 weeks 50, 100 mg/kg/day	↓ NF-κB, NLRP3 inflammasome adapter protein (NLRP3, ASC, cle-caspase-1) ↓ IL-1β, IL-18, CAMs (ICAM-1, VCAM-1) ↑ AMPK	Decreased serum TG and TC Foam cell expression	[84]
Baicalin (BC)	*Scutellaria baicalensis* Georgi	ApoE^−/−^ mice (15% fat and 0.25% cholesterol)	Oral administration 8 weeks 20, 50, 100 mg/kg/day	↓ TNF, Caspase-1 ↓ IL-1β, IL-18, CAMs (ICAM-1, VCAM-1) ↓ Mitochondria ROS, total ROS	Reduced aortic plaque	[85]
Corilagin (CL)	Various medicinal plants	ApoE^−/−^ mice, HFD	Oral administration every other day for 2 weeks 10, 20, 40 mg/kg	↓ Olfr2, Adcy3, NLRP3 Inflammasome assembly, GSDMD ↓ IL-1β, IL-18, TNF-a, M1 macrophage ↑ M2 macrophage	Decreased TC, TG and LDL-C Increased HDL-C	[86]
Curcumin (CU)	*Curcuma longa* L.	C57BL/6 mice, HFD	Oral administration 16 weeks 100 mg/kg/day	↓ NLRP3, ASC ↓ TNF-α, IL-1β ↓ NO, circulating endothelial cells	Reduction aortic plaque Decreased TC, TG and LDL-C Increased HDL-C	[87]
Dihydromyricetin (DHM)	*Ampelopsis grossedentata*	ApoE^−/−^ mice, HFD	Oral administration 14 weeks 50, 200 mg/kg/day	↓ TIM23, NK-κB p65, NLRP3, pro-caspase-1 ↓ IL-1β ↑ LC3-II/LC3I, PINK1/Parkin	Reduction aortic plaque Decreased TC, TG and LDL-C Increased HDL-C Promotion of mitophagy	[88]
Emodin (EM)	*Rheum palmatum* L.	ApoE^−/−^ mice, HFD	Oral administration 6 weeks 10, 20 mg/kg/day	↓ TLR4/MyD88 complex, NF-κB p65 nuclear translocation, NLRP3, GSDMD ↓ IL-1β, IL-18	Reduced atherosclerotic plaque areas	[89]
Polydatin (PD)	*Polygonum cuspidatum*	ApoE^−/−^ mice, HFD	Oral administration 8 weeks 50, 100, 200 mg/kg/day	↓ NLRP3, ASC, cle-caspase-1, GSDMD-N, Pyroptosis ↓ IL-1β, IL-18 ↑ Levels of autophagy (↓ p-mTOR/p62, ↑ LC3-II/LC3-I)	Reduced atherosclerotic plaque areas Stabilized vulnerable plaques Decreased TC, TG and LDL-C Increased HDL-C	[90]
Resibufogenin (RBG)	Toad venom	ApoE^−/−^ mice, HFD	Oral administration 8 weeks 1, 3, 5 mg/kg/day	↓ NLRP3, ASC, cle-caspase-1 ↓ M1 macrophage, IL-1β, IL18 ↑ M2 macrophage	Reduction aortic plaque Decreased TC, TG and LDL-C Increased HDL-C Inhibition of macrophage infiltration Promotion of mitophagy Direct binding to Cys-279 residue in NACHT domain	[91]
Salvianolic acid B (SAB)	*Salvia miltiorrhiza*	LDLR^−/−^ mice, HFD	IP injection 14 weeks 12.5, 25, 50 mg/kg/day	↓ NF-κB p65 nuclear translocation, NLRP3, Pyroptosis ↓ ROS production	Decreased TC, TG and LDL-C Decreased lipid deposition Reduced formation of atherosclerotic lesions	[92]
Scutellarin (SCU)	*Erigeron breviscapus* (Vant.) Hand.-Mazz.	ApoE^−/−^ mice, HFD	Oral administration 8 weeks 30 mg/kg/day	↓ NLRP3 inflammasome adapter protein(NLRP3, ASC) ↑ Autophagic flux	Foam cell expression Liver lipid accumulation mitigate Decreased TG Scutellarin OA induced plin2 in mRNA	[93]

↑, upregulation or activation; ↓, downregulation of inhibition. AMPK, AMP-activated protein kinase; cle-caspase, cleaved caspase; ApoE, Apolipoprotein E; mTOR, Mammalian target of rapamycin; NACHT, domain present in NAIP, CIITA, HET-E and TP1; NF-κB, Nuclear factor-kappa B; NLRP3, NOD-like receptor family pyrin domain containing 3; ROS, Reactive oxygen species; TLR4, Toll-like receptor 4.

## 4. Discussion

The synthesis of preclinical data presented in this review illuminates the formidable immunopharmacological potential of natural products in deactivating the NLRP3 inflammasome axis to combat atherosclerotic cardiovascular disease (ASCVD). In contrast to conventional pharmacological paradigms that singularly prioritize lipid reduction or rely on non-specific, broad-spectrum immunosuppression, natural product-derived inhibitors provide a highly sophisticated, multi-targeted approach capable of addressing the persistent residual inflammatory risk inherent in atherosclerosis [21,94,95]. While the clinical advent of statins and PCSK9 inhibitors has unequivocally revolutionized the management of dyslipidemia, epidemiological evidence confirms that aggressive LDL-cholesterol lowering alone fails to completely abrogate cardiovascular events. This clinical reality sharply highlights chronic vascular inflammation as a relentless driver of residual risk [30,31,34]. In this context, the NLRP3 inflammasome stands out as the pivotal molecular hub seamlessly linking metabolic dysregulation to the innate immune response, making its targeted inhibition by natural agents a highly promising frontier in vascular medicine [37,38,96].

The defining immunopharmacological advantage of natural products lies in their inherently pleiotropic mechanisms of action, allowing them to simultaneously recalibrate metabolic dysregulation and dampen hyperactive innate immune responses. Our comprehensive analysis reveals that these diverse compounds do not merely disrupt the final structural assembly of the NLRP3 inflammasome, but actively intercept the inflammatory cascade across multiple upstream and downstream pharmacological checkpoints. For instance, specific agents such as Apple Polyphenol Extract (APE), emodin, and baicalin effectively dismantle the requisite priming signal by crippling the TLR4/NF-κB signaling axis, thereby starving the cell of the transcriptional availability of NLRP3 and pro-IL-1β long before the inflammasome can even form [80,85,89]. Conversely, botanical derivatives like Crataegus aronia extract, Ginkgo Flavone Aglycone (GFA), and salvianolic acid B primarily target the activation signal. They achieve this by potently scavenging mitochondrial ROS and fortifying Nrf2/HO-1-mediated antioxidant defenses, an action that effectively neutralizes the severe oxidative stress required for NLRP3 assembly [81,82,89].

Furthermore, compounds including artemisinin, polydatin, and scutellarin demonstrate a remarkable capacity to reactivate stalled autophagic flux via the AMPK/mTOR pathway [84,90,93]. This pharmacological restoration of autophagy accelerates the selective clearance of severely damaged mitochondria and toxic inflammasome aggregates—a critical cellular housekeeping process termed mitophagy, which is characteristically impaired in advanced atherosclerosis [97]. Notably, recent mechanistic breakthroughs regarding Guizhitongluo Tablet (GZTLT) and resibufogenin reveal highly unique modes of action: GZTLT specifically antagonizes mechanosensitive Piezo1 channels to block calcium influx, while resibufogenin binds directly to the NACHT domain of the NLRP3 protein. These findings compellingly suggest that natural products can exert high-affinity, specific structural inhibition entirely comparable to rigorously designed synthetic small molecules [83,91]. This profound mechanistic diversity empowers natural products to exert synergistic therapeutic effects that may surpass the clinical efficacy of single-target drugs, while potentially offering a superior safety profile by preserving essential, baseline host defense mechanisms [40,41].

### 4.1. Challenges and Limitations in Translation

Despite these highly promising therapeutic trajectories, several critical translational barriers currently impede the clinical deployment of natural NLRP3 inhibitors. A paramount pharmacological challenge remains the notoriously poor bioavailability and unfavorable pharmacokinetic profiles characteristic of many polyphenolic compounds [41]. Bioactive substances such as curcumin, resveratrol derivatives, and various flavonoids frequently suffer from low aqueous solubility, rapid hepatic first-pass metabolism, and severely restricted gastrointestinal absorption. Consequently, these agents often yield sub-therapeutic plasma concentrations in vivo that fail to replicate the potent efficacy observed in controlled in vitro models [98,99,100]. For example, the absolute oral bioavailability of curcumin is documented to be less than 1%, a pharmacokinetic bottleneck that severely restricts its systemic clinical utility despite its indisputable anti-inflammatory activity [100].

Additionally, the rigorous standardization of these agents poses a substantial regulatory and scientific hurdle, particularly concerning complex botanical formulations like Guizhi Tongluo Tablet (GZTLT) or Agaricus bisporus (AB) extract [79,83]. Unavoidable variability in phytochemical composition—driven by geographic origins, seasonal harvesting shifts, and differing extraction methodologies—profoundly complicate quality control and make it exceedingly difficult to establish reliable dose–response relationships in clinical settings [101,102]. Furthermore, the vast majority of current preclinical efficacy data relies heavily on murine models, predominantly ApoE^−/−^ or LDLR^−/−^ mice [103]. While these genetically modified models yield invaluable mechanistic insights, they fail to fully replicate the complex architectural instability, spontaneous rupture events, and nuanced immune system characteristics defining human atherosclerosis, inevitably creating a potential translational gap [104,105]. Finally, the relatively low target selectivity of certain natural compounds raises valid concerns regarding “off-target” pharmacological effects at supraphysiological doses. For instance, high doses of certain polyphenols may interfere with essential cellular signaling pathways unrelated to the NLRP3 axis, or in the case of emodin, cause unintended gastrointestinal or hepatic responses. Such interactions necessitate rigorous, comprehensive toxicological evaluation and a deeper understanding of structure-activity relationships to refine target specificity before clinical application [106,107]. The key translational hurdles, potential off-target interactions, and proposed mitigation strategies for representative natural NLRP3 inhibitors discussed in this review are systematically summarized in Table 3.

Beyond pharmacokinetic issues, the economic viability of natural products often faces skepticism due to the challenges of securing robust patent protection for raw substances. Nevertheless, the potent inhibitory effects of these agents on the NLRP3 inflammasome provide a compelling scientific incentive for further drug development. These intellectual property hurdles are increasingly being addressed through patentable innovations, including standardized synergistic combinations and advanced drug delivery systems (DDS). Such strategies enable the industry to secure commercial exclusivity while harnessing the therapeutic potential of natural scaffolds for clinical application.

### 4.2. Future Perspectives and Clinical Directions

To successfully bridge the translational gap between the bench and the bedside, and to fully harness the immunomodulatory power of these natural agents, future research endeavors must strategically pivot toward several key areas. To circumvent persistent pharmacokinetic barriers, the integration of advanced drug delivery systems (DDS) is rapidly emerging as a critical, transformative strategy [109,110,111]. The engineering of nanotechnology-based delivery platforms—encompassing lipid-based liposomes, polymeric nanoparticles, and bio-inspired exosomes—has demonstrated immense promise in dramatically enhancing the biochemical stability, aqueous solubility, and targeted spatial delivery of natural compounds directly to vulnerable atherosclerotic plaques [112,113,114].

Recent cutting-edge studies have demonstrated that biomimetic, macrophage-targeted nanoparticles can substantially increase the localized accumulation of anti-inflammatory agents within the inflamed vascular wall, thereby maximizing site-specific therapeutic efficacy while simultaneously minimizing the risk of systemic side effects [115,116]. Concurrently, modern medicinal chemistry approaches must be actively employed to structurally modify the core molecular scaffolds of these bioactive natural products, optimizing their fundamental drug-like properties. The deliberate structural optimization of lead compounds—such as the synthesis of novel analogs of artemisinin or emodin—could remarkably enhance both their systemic bioavailability and their direct binding affinity to the NLRP3 receptor [108,117].

Moreover, the most viable clinical application of these immunomodulators will likely materialize as a highly synergistic combination therapy. Investigating the strategic co-administration of natural NLRP3 inhibitors alongside current standard-of-care pharmacotherapies, specifically statins or PCSK9 inhibitors, represents a highly pragmatic and forward-thinking clinical strategy [118,119]. Such dual-therapy regimens could deliver profound additive benefits by simultaneously reducing atherogenic LDL-cholesterol levels while attenuating localized vascular inflammation, thereby addressing the residual inflammatory risk identified in major clinical trials such as CANTOS and COLCOT [24,26,120]. Ultimately, the clinical validation of these compelling preclinical findings demands rigorous, well-powered, randomized, placebo-controlled clinical trials [121]. Crucially, these future trials must not only evaluate hard cardiovascular endpoints but also systematically incorporate specific, quantifiable circulating biomarkers of inflammasome activity—including serum IL-1β, IL-18, and active caspase-1 levels—to conclusively prove their mechanism-based therapeutic efficacy in human cohorts [95].

## 5. Conclusions

Atherosclerotic cardiovascular disease unequivocally remains a primary driver of global mortality, urgently necessitating the development of novel therapeutic strategies capable of mitigating the residual inflammatory risk that persists despite achieving optimal lipid-lowering targets. In this pathological landscape, the NLRP3 inflammasome has firmly established itself as a central immunometabolic hub, actively driving the chronic, destructive vascular inflammation intrinsically associated with atherosclerosis. This review has comprehensively synthesized the robust, emerging preclinical evidence supporting the therapeutic efficacy of natural product-derived inhibitors, systematically categorizing them into highly synergistic complex extracts and mechanistically precise single bioactive compounds. The accumulated data compellingly demonstrate that these natural agents masterfully inhibit NLRP3 inflammasome activation through highly coordinated pleiotropic mechanisms. These include the potent suppression of upstream TLR4/NF-κB priming, the efficient scavenging of pathological mitochondrial ROS, the restoration of autophagic flux via AMPK/mTOR signaling, and the direct structural interference with the inflammasome assembly complex itself.

Although the successful clinical translation of these immunopharmacological findings currently confronts distinct hurdles—namely low oral bioavailability, phytochemical standardization challenges, and the inherent limitations of murine models—the strategic integration of state-of-the-art nanomedicine delivery technologies and targeted structural optimization offers a highly viable roadmap to overcome these obstacles. Natural products possess a unique, powerful therapeutic advantage due to their multi-targeted pharmacological effects and generally favorable toxicological safety profiles when compared to synthetic, single-target immunosuppressants. Consequently, future research initiatives must rigorously prioritize the clinical validation of these compounds within well-designed human trials, particularly targeting high-risk patient populations exhibiting elevated systemic inflammatory markers. Ultimately, natural NLRP3 inflammasome inhibitors represent a highly transformative, next-generation therapeutic class that, upon successful clinical development, possesses the profound potential to significantly alleviate the global burden of atherosclerotic cardiovascular disease by directly striking at the root cause of vascular inflammation.

## Figures and Tables

**Table 3:** Summary of translational challenges and strategic solutions for representative natural NLRP3 inhibitors.

Representative Compound	Translational Hurdles (Bioavailability/Metabolism)	Key Enzymes & Potential Off-Targets	Synergistic & Overcoming Strategies	Ref.
Curcumin	Extremely low (<1%); Rapid 1st-pass	CYP3A4, UGTs; DNA damage at high doses	Synergy w/Piperine; Targeted nano-micelles	[41,100]
Artemisinin	Low aqueous solubility	CYP3A4; Neurotoxicity (supraphysiological dose)	Synergy w/ROS scavengers; Structural analogs	[84,108]
Emodin	Rapid hepatic metabolism	CYP1A2; Hepatotoxicity/Laxative effects	Combination w/P2X7R antagonists; Nanoemulsions	[89,109]
Baicalin	Rapid gut/hepatic metabolism	UGTs; Potential GI discomfort	Potentiates Statins; Cyclodextrin complexes	[85]
Resibufogenin	Rapid clearance	Minimal off-targets due to high NACHT specificity	Combination w/low-dose anti-inflammatories	[91]

## Data Availability

No new data were created or analyzed in this study. Data sharing is not applicable to this article.
